# Egg envelopes and cuticle renewal in *Porcellio* embryos and marsupial mancas

**DOI:** 10.3897/zookeys.176.2418

**Published:** 2012-03-20

**Authors:** Polona Mrak, Nada Žnidaršič, Magda Tušek-Žnidarič, Waltraud Klepal, Daniela Gruber, Jasna Štrus

**Affiliations:** 1 Department of Biology, Biotehnical faculty, University of Ljubljana, Večna pot 111, SI-1000 Ljubljana, Slovenia; 2 Department of Ultrastructural Research, University of Vienna, Althanstrasse 14, A-1090 Vienna, Austria

**Keywords:** Chorion, vitelline membrane, cuticle, molting, ontogenetic development, terrestrial isopods

## Abstract

An important adaptation to land habitats in terrestrial isopod crustaceans is development of embryos in a fluid-filled female brood pouch, marsupium. The study brings insight into the structure and protective role of egg envelopes and cuticle renewal during ontogenetic development of *Porcellio* embryos and marsupial mancas*.* Egg envelopes cover embryos, the outer chorion until late-stage embryo and the inner vitelline membrane throughout the whole embryonic development. Egg envelopes of *Porcellio* have relatively simple ultrastuctural architecture compared to *Drosophila* egg envelopes. Exoskeletal cuticle is produced in late embryonic development by hypodermal cells of the embryo and is renewed in further development in relation to growth of developing embryos and mancas. Cuticle structure and renewal in prehatching late-stage embryos and marsupial mancas exhibit main features of cuticle in adults. Epicuticle is thin and homogenous. The characteristic arrangement of chitin-protein fibers and the dense distal layer in exocuticle are hardly discernible in prehatching embryo and distinct in marsupial mancas. Endocuticle consists of alternating electron dense and electron lucent sublayers and is perforated by pore canals in both stages. Differences from adult cuticle are evident in cuticle thickness, ultrastructure and mineralization. Signs of cuticle renewal in prehatching embryo and marsupial mancas such as detachment of cuticle from hypodermis, partial disintegration of endocuticle and assembly of new cuticle are described.

## Introduction

The unique feature of embryonic development in isopod and amphipod crustaceans (Peracarida) is its location in the brood pouch on the ventral side of female body (marsupium). The marsupium has likely been of a great adaptive significance in the colonization of land by crustacean species as it allows embryonic development to occur in aqueous environment within the protected chamber (Hornung 2011, Warburg 2011). Two main types of marsupium are distinguished in Oniscidea, the amphibian or open type and the terrestrial or closed type marsupium (Hoese and Janssen 1989). In the former, marsupium is partly open and water from the external environment can pass into the marsupium. In the closed type, marsupium is a watertight structure, provisioned only with fluid from the mother. Marsupial fluid is persistently osmotic and ionic regulated, probably via transporting epithelia of segmental cotyledons, which hang down from the overlying sternites (Surbida and Wright 2001). Depending on the level of maternal control of the marsupial environment, osmotic tolerance of the embryos and marsupial mancas is of adaptive importance. Protective envelopes between embryo/manca and marsupial fluid function against potential physiological stresses, including osmotic and ionic variation and desiccation in marsupial environment (Charmantier and Charmantier-Daures 2001, Surbida and Wright 2001). Ontogenetic developmentof terrestrial isopod crustacean *Porcellio scaber*,from released fertilized eggs to embryos and marsupial mancas, occurs in the terrestrial type marsupium. Intramarsupial development of *Porcellio scaber* lasts approximately 35 days (Milatovič et al. 2010), and during development individuals in different stages are coated by different protective envelopes - egg envelopes (chorion and vitelline membrane) and cuticle. During growth of embryos and mancas egg envelopes are shed and cuticle is renewed.

Two egg envelopes, the outer chorion and the inner vitelline membrane, are produced during oogenesis by the somatic follicle cells of the female reproductive system and cover the embryo until the transition to the late-stage embryo and hatching, respectively. Arthropods evolved egg envelopes of different morphologies and complexities as a consequence of embryonic development in different environments. The data on egg envelopes (chorion and vitelline membrane) structure derive mainly from the studies on the fruit fly *Drosophila melanogaster*, as this model species offers wide opportunities for genetic studies (Margaritis et al. 1980, Jagadeeshan and Singh 2007), while there are no data on isopod chorion and vitelline membrane ultrastructure. In contrast to *Porcellio scaber* embryonic development in the female protected environment*,* *Drosophila* embryonic development takes place independently of the female. The egg envelopes of *Drosophila*, especially the chorion, are structurally complex and their proteins show signs of evolving under selection by ecological factors. At the morphological level, differences in egg envelopes surfaces between specialists and generalists of *Drosophila* species were found, particularly in the surface ridges and surface porosity (Jagadeeshan and Singh 2007).

Cuticle is the innermost protective layer, formed later during intramarsupial development and is produced by hypodermal cells of the embryo. In adult arthropods the cuticle is a complex hierarchically structured extracellular matrix, consisting of chitin, proteins and lipids, hardened mostly by mineralization in crustaceans in contrast to insect cuticle which is only sclerotized. It comprises the distal epicuticle, the exocuticle in the middle and the proximal endocuticle. Several reports were published on cuticle structure in adult isopods, mostly in *Porcellio scaber* (Price and Holdich 1980, Štrus and Compere 1996, Ziegler 1997, Glötzner and Ziegler 2000, Štrus and Blejec 2001, Hild et al. 2008, Hild et al. 2009). The thin and non-calcified epicuticle is composed mainly of lipoproteins and consists of thinner 5-layered outer epicuticle and thicker inner epicuticle. The exocuticle, comprising sublayers of chitin–protein fibers arranged in characteristic pattern and the endocuticle, consisting of lamellar chitin–protein sublayers, are calcified. In addition, the thin non-calcified membranous layer lies between the endocuticle and the epithelial cells. The ultrastructure and composition of the cuticle in isopod embryos and marsupial mancas have not been studied in detail, while cuticle structure is well described in *Drosophila melanogaster* embryos (Locke 2001, Payre 2004, Moussian et al. 2006, Moussian 2010). Fully formed cuticle in *Drosophila* embryo is organized in distinct horizontal layers. The distal lipoprotein epicuticle is subdivided in the outer thin epicuticle (cuticulin layer) and the inner thick epicuticle and the proximal chitin-protein procuticle consists of several lamellae.

Cuticle renewal is related to growth in arthropods. In crustaceans molting frequently recurs during adult life. Isopod crustaceans molt in two phases, separately molting posterior and anterior parts of the body. Molt cycle begins with premolt stage, when remarkable morphological changes of the integument occur. The old cuticle separates from the underlying epithelium (apolysis). Epithelial cells secrete a new cuticle, starting with the epicuticle and followed by pre-ecdysal exocuticle. The old and the new cuticles are separated by an extracellular compartment, the ecdysal space, containing different material involved in cuticle renewal. At molting the old cuticle is shed and the new cuticle is further produced, forming post-ecdysal endocuticle. Postmolt stage is marked by soft body surface with progressive hardening of the exoskeleton until the intermolt stage.

In this study we present new data on the ultrastructural architecture of egg envelopes, including chorion and vitelline membrane, and on the ultrastructural characteristics of cuticle renewal in embryos and marsupial mancas of isopod crustaceans *Porcellio scaber* and *Porcellio dilatatus*. Comparison of envelopes structure in terrestrial crustaceans and insects will bring new insights into the protective role of egg envelopes and cuticle renewal in developing embryos of these two terrestrial arthropod groups with different developmental strategies.

## Methods

Animals were maintained and bred in a laboratory culture. Staging system of *Porcellio scaber* ontogenetic development, based on morphological characteristics of embryos and marsupial mancas, was used in this study (Milatovič et al. 2010). Embryos of *Porcellio dilatatus* were used in scanning electron microscopic studies of egg envelopes.

**Figure 1.**
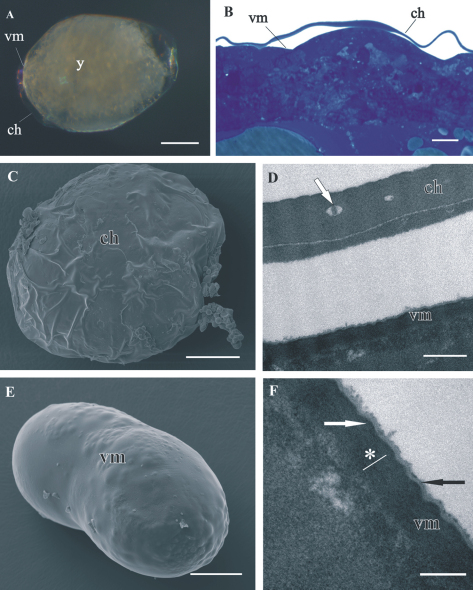
Structure of distal chorion (**ch**) and proximal vitelline membrane (**vm**), covering *Porcellio scaber* **A,** **B, D, F** and *Porcellio dilatatus* **C, E** early-stage embryo. **A** The early-stage embryo with large amount of yolk (**y**) and no visible limb buds. **B** Semithin section of the embryo peripheral region. Chorion is separated from the embryo surface. The vitelline membrane is closely apposed to the embryo surface. **C** SEM micrograph of the early-stage embryo. The outer egg envelope, chorion, is visible. **D** TEM micrograph of one-layered chorion, including electron lucent “lacunae” (white arrow). There is a layer of artificially spilt yolk underneath the chorion. **E** SEM micrograph of the early-stage embryo. Chorion is artificially removed and the inner egg envelope, vitelline membrane, is exposed. **F** TEM micrograph of vitelline membrane, composed of three layers: main proximal homogenous layer (*), thin middle electron dense layer (white arrow) and superficial corrugated lucent layer (black arrow). Bars: **A, C, E** 200 µm; **B** 10 µm; **D** 0.5 µm; **F** 200 nm.

**Figure 2.**
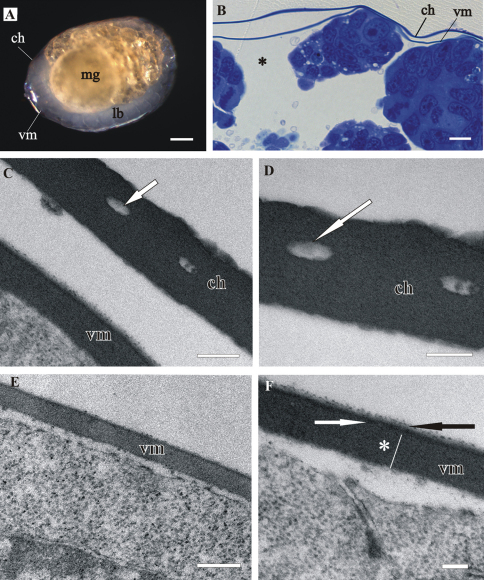
Structure of distal chorion (**ch**) and proximal vitelline membrane (**vm**), covering *Porcellio scaber* mid-stage embryo. **A** The mid-stage embryo with visible limb buds (**lb**) and midgut glands primordia (**mg**). **B** Semithin section of the embryo peripheral region. Chorion is separated from the embryo surface. The vitelline membrane is slightly detached from the embryo cells; * - a wider space between embryo surface and vitelline membrane. **C, D** TEM micrographs of one-layered chorion, including electron lucent “lacunae” (white arrow). **E, F** TEM micrographs of vitelline membrane, composed of three layers: main proximal homogenous layer (*), thin middle electron dense layer (white arrow) and superficial corrugated lucent layer (black arrow). Bars: **A** 200 µm; **B** 10 µm; **C, E** 0.5 µm; **D, F** 200 nm.

**Figure 3.**
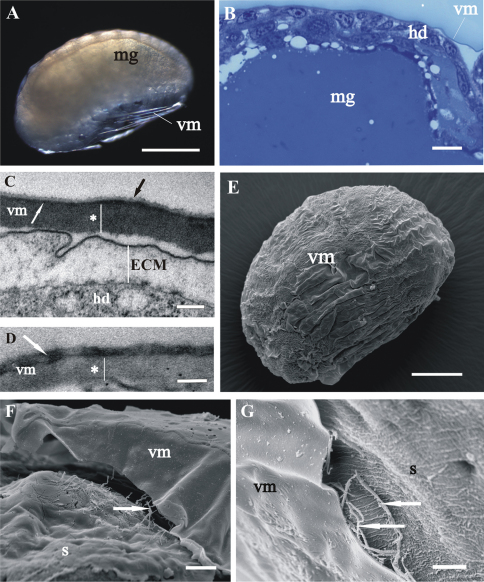
Structure of vitelline membrane (**vm**), covering *Porcellio scaber* **A–D** and *Porcellio dilatatus* **E–G** late-stage embryo. **A** Ventrally bent late-stage embryo, yolk is completely enclosed into the midgut glands (**mg**). **B** Semithin section of the embryo peripheral region. Vitelline membrane is slightly detached from the hypodermis (**hd**). **C, D** TEM micrographs of the vitelline membrane in osmicated specimen **C** and in non-osmicated specimen **D** Main proximal homogenous layer (*), thin middle electron dense layer (white arrow) and superficial corrugated lucent layer (black arrow). Hypodermis is covered with an extracellular matrix (ECM). **E** SEM micrograph of the late-stage embryo surrounded by vitelline membrane. **F, G** SEM micrographs of the late-stage embryo surface area. The vitelline membrane is artificially slit and fibers (arrows) between the outer embryo surface, covered with an extracellular matrix (s), and the vitelline membrane are exposed. Bars: **A** 500 µm; **B, F** 10 µm; **C, D** 200 nm; **E** 200 µm; **G** 5 µm.

**Figure 4.**
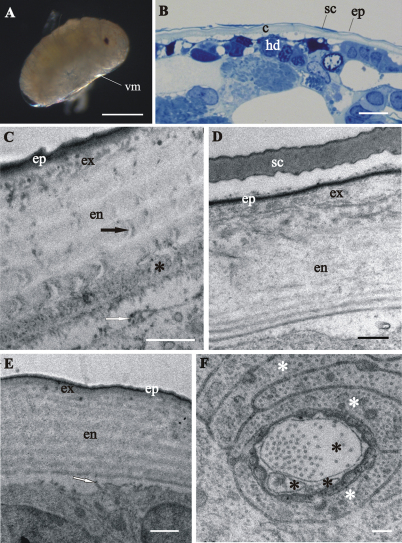
Cuticle structure and renewal in *Porcellio scaber* prehatching late-stage embryo. **A** Swelled embryo inside the vitelline membrane (**vm**), prior to hatching. **B** Semithin section of the embryo peripheral region. The vitelline membrane is artificially removed. Clearly discernible exoskeletal cuticle (**c**), detached from the underlying hypodermis (**hd**). **C, D, E** TEM micrographs of exoskeletal cuticle in different regions of the same specimen, composed of three principal layers: the outermost thin electron dense epicuticle (**ep**), the middle exocuticle (**ex**) and the innermost endocuticle with several sublayers (**en**). The micrographs show features of cuticle renewal: cuticle detachment from the hypodermis, partial disintegration of proximal portion of endocuticle (*) and irregularly arranged electron dense particles on outer apical plasma membrane surface (white arrows). Pore canals (black arrow) in the endocuticle consist of electron lucent central part and electron dense margins **C**. Cuticular scales (**sc**) are fully elaborated and the exocuticle has the characteristic pattern of chitin-protein fibers arrangement **D**. Exocuticle is hardly discernible **E**. **F** TEM micrograph of completely structured sensillum transverse section in the hypodermis. Dendritic outer segments (*) and enveloping cells (white *). Bars: **A** 500 µm; **B** 10 µm; **C, E** 1 µm; **D** 0.5 µm; **F** 200 nm.

**Figure 5.**
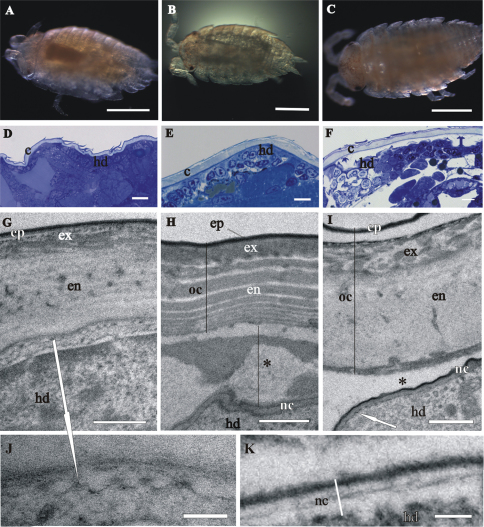
Cuticle structure and renewal in *Porcellio scaber* marsupial mancas. **A** The early-stage marsupial manca, immediately after hatching **B** The mid-stage marsupial manca **C** The late-stage marsupial manca, just prior to release from the marsupium **D–F** Semithin sections of the manca peripheral region in the early-stage marsupial manca **D** in the mid-stage marsupial manca **E** and in the late-stage marsupial manca **F**. Cuticle (**c**), overlying the hypodermis (**hd**), becomes progressively more similar to adult cuticle. **G–K** TEM micrographs of exoskeletal cuticle in the early-stage marsupial manca **G, J** in the mid-stage marsupial manca **H** and in the late-stage marsupial manca **I, K** Three main layers are distinguished: epicuticle (**ep**), exocuticle (**ex**) and endocuticle (**en**). The micrographs show morphological characteristics of cuticle renewal: detachment of the old cuticle (**oc**) from the hypodermis, ecdysal space (*) between the detached cuticle and the newly forming cuticle (**nc**) and partial degradation of the old cuticle **H, I** protrusions with electron dense tips (white arrows) on apical surfaces of hypodermal cells **G, I, J**. The new cuticle consists of two layers, external electron dense epicuticle and internal electron lucent procuticle **H, I, K**. Helicoidal chitin-protein fibers arrangement is discernible in some regions of late-stage marsupial manca **K**. Bars: **A–C** 500 µm; **D–F** 10 µm; **G–I** 1 µm; **J, K** 200 nm.

The embryos and marsupial mancas of different developmental stages are shown in Figures 1A, 2A, 3A, 4A and 5A-C in the Results. The term early-stage embryo is used for embryos with large amount of yolk mass in the central part and no visible limb buds. A mid-stage embryo has visible developing limb buds and two midgut glands primordia, which enclose yolk. After bending ventrally and shedding of chorion, embryos are termed late-stage embryos. Prior to hatching swelled embryo inside the vitelline membrane is described as a prehatching late-stage embryo. When late embryos hatch from the vitelline membrane they become marsupial mancas. The progress of development of *Porcellio scaber* marsupial mancas is characterized by the following morphologically discernible modifications: reduction of the midgut glands size due to yolk consumption, increase in exoskeleton pigmentation, enlargement of body size and pronounced locomotion (Wolff 2009, Milatovič et al. 2010). In previous studies stages of marsupial mancas were not precisely determined. For this reason and according to the morphological characteristics listed above, we determined three sequential developmental stages of marsupial mancas, early-stage, mid-stage and late-stage marsupial mancas. The term early-stage manca is used for 1.5 – 1.6 mm long mancas with no or very little locomotion inside the marsupium, with scarce chromatophores on the body surface and with the midgut yolk extending into the pleon. Mid-stage mancas are 1.7 - 1.8 mm long, with darker pigmentation on the head region and tergites and with the midgut yolk only partly extending into the pleon. Late-stage mancas are 1.9 - 2.0 mm long, with pronounced locomotion of the whole body and pereopods. The yolk in the midgut extends only to the end of the pereon.

Embryos and mancas at different stages of development were isolated from the marsupium and fixed in 2.5% glutaraldehyde in 0.1 M cacodylate buffer (pH 7.2). Prior to fixation, the egg envelopes of embryos were either carefully perforated with a thin needle or removed. After washing in cacodylate buffer, the samples were postfixed in 1% osmium tetroxide for 2 hours, washed again and dehydrated in a graded series of ethanol.

Embryos and mancas of *Porcellio scaber* for light microscopy (LM) and transmission electron microscopy (TEM) were embedded in Agar 100 resin. Prior to embedding, mancas were perforated with a thin needle for better infiltration of resin. Semithin sections were made with a glass knife, stained with Azure II - Methylene Blue and imaged by Zeiss AxioImager Z.1 light microscope, equipped with a HRC Axiocam camera. Ultrathin sections were made with a Reichert Ultracut S ultramicrotome (Leica), contrasted with 4% uranyl acetate for 10 minutes and 10% lead citrate for 5 minutes and inspected with a Philips CM100 transmission electron microscope, equipped by BioScan 792 camera (Gatan).

After dehydration in a graded series of ethanol and in acetone, *Porcellio dilatatus* specimens for scanning electron microscopy (SEM) were transferred into hexamethyldisilazane (HMDS) to perform chemical drying. Mounted specimens were coated with gold and observed with scanning electron microscopes (Philips XL20 and Philips XL30).

For histochemical detection of calcified tissue, mancas of *Porcellio scaber* were isolated from the marsupium and fixed in 3.7% formaldehyde in 0.1 M cacodylate buffer (pH 7.2). Specimens were washed in cacodylate buffer and embedded in tissue freezing medium (Jung). Transversal sections (10 µm) were cut with a Leica CM1850 cryostat at –18°C and stained with Alizarin red S solution in 0.2 M Trihydroxylmethyl aminomethane (Tris) - HCl buffer (pH 9). Cuticle ofadult *Porcellio scaber* was used as a positive control. Sections were imaged by Zeiss AxioImager Z.1 light microscope, equipped with a HRC Axiocam camera.

## Results

Ontogenetic development of *Porcellio scaber* in the marsupium, from released fertilized eggs to marsupial mancas, was recently described morphologically (Wolff 2009, Milatovič et al. 2010), but the issue of protective envelopes was not addressed specifically. We present here the ultrastructural architecture of chorion, vitelline membrane and cuticle in embryos and marsupial mancas of isopod crustaceans *Porcellio scaber* and *Porcellio dilatatus*.

### Ultrastructure of chorion and vitelline membrane

Both egg envelopes, distal chorion and proximal vitelline membrane, surround early-stage (Figs 1A, B, C, E) and mid-stage embryos (Figs 2A, B). Chorion has a similar appearance in both stages. It is a one-layered envelope, separated from the embryo surface and is approximately 500 nm thick. Ultrastructurally chorion consists of an electron dense matrix with sparse electron lucent “lacunae” (Figs 1D, 2C, 2D).

The vitelline membrane surrounds embryos throughout the whole developmental period (Figs 1A, 1B, 1E, 2A, 2B, 3A, 3B, 3E, 4A). It maintains the same thickness of approximately 200 nm from early-stage till late-stage embryo. It is closely apposed to the embryo surface in early-stage embryos (Figs 1B, D, F), while it is slightly detached from the embryo surfaces of mid-stage embryos (Figs 2B, E, F) and late-stage embryos (Figs 3B, C, F, G). Above the embryo limb buds a wider space appears between embryo surface and vitelline membrane due to intense cell rearrangement during limb buds formation (Fig. 2B). The vitelline membrane consists of a thick proximal homogenous electron dense matrix superposed by a thin middle electron dense layer and a superficial corrugated lucent layer (Figs 1F, 2E, 2F, 3C). In non-osmicated specimens of late-stage embryo, the main thick layer is evidently lighter and superficial layer is not discerned (Fig. 3D). Between the outer embryo surface and the vitelline membrane of late-stage embryo a network of fibers was observed by SEM, presumably functioning as connective elements (Figs 3F, G).

### Structure and renewal of cuticle

In late-stage embryo the embryo surface is covered with a homogenous extracellular matrix (Figs 3C, F, G). The prehatching late-stage embryo is the earliest developmental stage, in which the overall ultrastructural architecture of the cuticle is similar to the adult crustacean cuticle and first morphological evidence of cuticle renewal is evident. Cuticle is from 2 to 3 µm thick, which is significantly thinner than in adults. It is composed of three layers, the outermost thin electron dense epicuticle, the middle exocuticle and the innermost endocuticle (Figs 4C, D, E). Cuticular scales are fully elaborated (Fig. 4B, D). Several transverse sections of completely structured sensilla are observed in the hypodermis (Fig. 4F). Dendritic outer segments and enveloping cells are clearly differentiated. The characteristic pattern of chitin-protein fibers arrangement in the exocuticle is resolved in some regions and hardly discernible in other regions of the same specimen. The endocuticle is subdivided in several electron dense sublayers alternating with electron lucent sublayers (Figs 4C, D, E). In some regions pore canals are visible running through the endocuticle, consisting of electron lucent central part and electron dense margins (Fig. 4C). Several morphological features in prehatching late-stage embryo show the exoskeletal cuticle renewal already in this stage of development: partly disintegrated proximal portion of endocuticle in some regions (Fig. 4C); cuticle detachment from the hypodermis (Figs 4B, C, D, E); rough apical plasma membranes of hypodermal cells and irregularly arranged electron dense particles on their outer surface (Figs 4C, E).

Next, we present evidence of cuticle renewal in different stages of marsupial mancas, namely: (I) early-stage marsupial manca, immediately after hatching (Fig. 5A); (II) mid-stage marsupial manca (Fig. 5B) and (III) late-stage marsupial manca, just prior to release from the marsupium (Fig. 5C). In the marsupial mancas the cuticle has similar architecture as the cuticle of prehatching embryo (Figs 5D–I). The main difference is the more pronounced characteristic chitin-protein pattern of exocuticle with a dense distal layer in marsupial mancas in comparison to embryo (Figs 5G, I). The micrograph of the late-stage marsupial manca cuticle displays well-formed pattern of exocuticle, resembling the adult helicoidal exocuticle structure and with a dense distal layer (Fig. 5I). The endocuticle of marsupial mancas is perforated by pore canals. The thickness of the marsupial manca cuticles is up to 3 µm. Morphological characteristics of cuticle renewal are observed in all three stages of marsupial mancas (Figs 5G-K). Apolysis, detachment of the old cuticle from the hypodermis is clearly visible in several stages (Figs 5F, H, I). The detached cuticle is partly degraded and much thinner in certain regions of the same specimen. The ecdysal space between the detached cuticle and the newly forming cuticle is also well evident and it contains homogenous electron dense material in some specimens (Fig. 5H), while in others it appears devoid of electron dense material (Fig. 5I). The newly assembling cuticle, covering hypodermal cells, consists of two layers, a thin electron dense external layer - epicuticle and an electron lucent procuticle (Figs 5H, I, K). In some regions of late-stage marsupial manca the procuticle appears homogenous (Fig. 5I), while in other regions helicoidal chitin-protein fibers arrangement is clearly discernible (Fig. 5K). The surface of the new cuticle is slightly (Fig. 5H) or highly wrinkled (Fig. 5I). Protrusions with electron dense tips are formed on apical surfaces of hypodermal cells. These characteristic features of cuticle synthesis are clearly visible underneath the assembling cuticle in all stages of marsupial mancas (Figs 5G, I, J).

**Figure 6.**
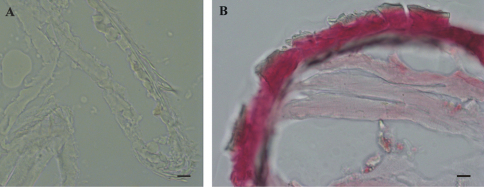
Histochemical reaction for calcium – Alizarin red S. **A** No reaction in *Porcellio scaber* late marsupial manca **B** Positive control (red-pink) in adult *Porcellio scaber* cuticle. Bars: 10 µm.

The results of histochemical reaction with Alizarin red S for calcified tissues localization indicate that the cuticle of marsupial mancas is not strongly mineralized (Fig. 6A) in comparison to adult cuticle (Fig. 6B). The ventral calcium deposits, characteristic for adult premolt animals, were not observed in molting marsupial mancas.

**Figure 7.**
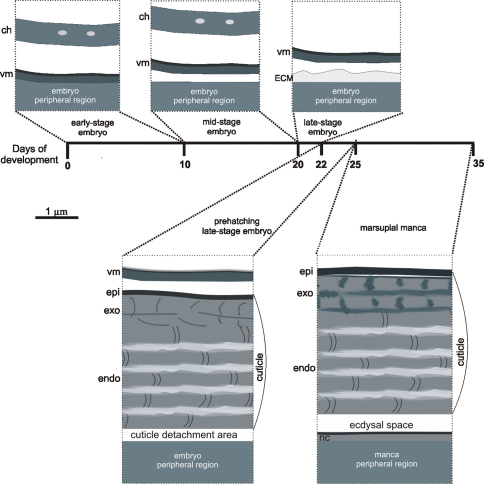
Schematic representation of different protective envelopes, coating *Porcellio scaber* embryos and marsupial mancas during development (lasting 35 days), namely egg envelopes (chorion and vitelline membrane) and exoskeletal cuticle. During growth of embryos and mancas egg envelopes are shed and cuticle is renewed. **ch** – chorion; **vm** – vitelline membrane; **ECM** – extracellular matrix; **epi** – epicuticle; **exo** – exocuticle; **endo** – endocuticle; **nc** – newly assembling cuticle.

### Discussion

The structure of egg envelopes and the ultrastructural architecture of the exoskeletal cuticle in *Porcellio* embryos and marsupial mancas share some common features with those described in other arthropods, but there are also some significant differences.

In comparison to egg envelopes of *Drosophila* eggs from ovaries, described byMargaritis et al. (1980), the egg envelopes of *Porcellio* embryos are thinner and have considerably less complex ultrastructure. Chorion of *Porcellio* is approximately two times thinner than chorion of *Drosophila*. The chorion of *Drosophila* eggs is differentiated into several layers, a thin innermost chorionic layer, a complex fenestrated endochorion and a fibrous exochorion,while *Porcellio* chorion in embryos consists of a single homogenous layer. Electron lucent “lacunae” observed in the *Porcellio* chorion resemble the cavities in the inner part of the *Drosophila* chorion. These complex cavities are thought to be air-filled in laid eggs and involved in respiration (Margaritis et al. 1980). Study of embryo tolerance to physiological stresses in terrestrial isopod *Armadillidium vulgare* shows that the chorion has low permeability to water and solutes and contributes to the very high tolerance to osmotic stress of early-stage embryo (Surbida and Wright 2001). The vitelline membrane of *Porcellio* embryos also appears thinner, but has a similar ultrastructure as *Drosophila* eggs vitelline membrane. Our results indicate that the osmiophilic main proximal layer and the thin superficial layer of *Porcellio* vitelline membrane differ in composition from the middle electron dense layer. The thin superficial layer may correspond to a similar thin layer above vitelline membrane in *Drosophila* egg (Margaritis et al. 1980). The authors presume that this layer in *Drosophila* consists of wax, functioning to reduce water loss. This is not expected for *Porcellio* embryos since they are less vulnerable to desiccation due to their development in aqueous environment. The comparison of egg envelopes ultrastructure in *Porcellio* embryos and in *Drosophila* eggs reveals several differences. We consider these dissimilarities the consequence of different environments of embryonic development in *Porcellio* embryos which develop in a protected fluid-filled maternal brood pouch and in *Drosophila* embryos which are directly exposed to external environment during development. Here we also report on a network of fibers between embryo surface and vitelline membrane in late-stage embryo. We presume that these fibers function as connective elements between embryo and envelope, but no comparative data on vitelline membrane attachment were found in the literature.

We show here that the late embryo is already covered with a homogenous extracellular matrix, possibly first cuticle. In the prehatching late-stage embryo of *Porcellio scaber* the exoskeletal cuticle has already main features of the adult crustacean cuticle, but minor differences are evident. It is composed of three principal layers - epicuticle, exocuticle and endocuticle. Endocuticle shows the typical arrangement of chitin lamellae in sublayers which are not so distinct as in adult cuticle. The characteristic pattern of chitin-protein fibers arrangement in the exocuticle of adults is not discernible in exocuticle of the prehatching late-stage embryo. A distal layer, similar to the dense distal exocuticular layer, described in adult isopods (Hild et al. 2009, Huber and Ziegler 2011, Seidl and Ziegler 2011), is not observed in the cuticle of prehatching embryo. Sensilla in hypodermis are already very elaborated and have similar ultrastructure as tricorn sensilla described in adult *Porcellio scaber* (Ziegler and Altner 1995). Previous studies of cuticle differentiation during embryonic development of *Drosophila* *melanogaster* and *Parhyale* *hawaiensis* show that cuticle ultrastructure in last stage embryo likewise resembles adult cuticle ultrastructure, with typical helicoidal arrangement of chitin lamellae in procuticle and several-layered epicuticle (Locke 2001, Moussian et al. 2006, Havemann et al. 2008, Moussian 2010). Our observations of hypodermal cells in the prehatching late-stage embryo are in agreement with those described in *Drosophila*, as they are both flattened, with centrally placed nucleus, scarce organelles and connected by septate junctions (Moussian et al. 2006). During development of *Porcellio scaber* marsupial mancas cuticle becomes progressively more similar to adult cuticle, particularly regarding the characteristic pattern of chitin-protein fibers in exo- and endocuticle, which is more and more explicit. In the distal portion of the exocuticle an electron dense layer is clearly resolved. It could correspond to the dense distal exocuticular layer, described in several adult isopods (Hild et al. 2009, Huber and Ziegler 2011, Seidl and Ziegler 2011). Several authors report on possibility of exoskeleton calcification in marsupial mancas. Inferred only by increased total calcium concentration of *Armadillidium vulgare* late-stage marsupial manca, Ouyang and Wright (2005) suggest that cuticle calcification starts in this stage. Surbida and Wright (2001) presume that wide osmotic tolerance of *Armadillidium vulgare* marsupial mancas is a consequence of calcification of their cuticle. Havemann et al. (2008) report on cuticle calcification after hatching of amphipod *Parhyale hawaiensis* embryo, but it is not known which larval stage was observed. Our research, using histochemical approach to localize calcified tissue, indicates that the exoskeletal cuticle of *Porcellio scaber* marsupial mancas is not strongly, if at all mineralized. It could be possible that amorphous calcium carbonate was dissolved during preparation, since it has relatively high solubility.

Next, the issue of cuticle renewal during intramarsupial development was addressed in this study. Partly disintegrated proximal endocuticle and cuticle detachment from the underlying hypodermis indicate that cuticle renewal takes place already in the prehatching embryonic stage. Prehatching embryo is thus the earliest stage of *Porcellio scaber* development, where renewal of exoskeleton, i.e. initiation of molting, was observed so far. These results are in agreement with the previous observation of apolysis on appendage tips in late-stage embryo of *Porcellio scaber* (Milatovič et al. 2010). Apolysis and cuticle disintegration were not observed in the studies of cuticle structure during embryonic development in insect *Drosophila melanogaster* and amphipod crustacean *Parhyale hawaiensis* (Locke 2001, Moussian et al. 2006, Havemann et al. 2008, Moussian 2010). We observed that the old cuticle is detached from the hypodermis and the new cuticle is produced in marsupial mancas of different stages, which is a sign of premolt. Several similarities and differences with respect to molting process in adult isopods are described. Similarities in apical protrusions of hypodermal cells during cuticle synthesis, and appearance of ecdysal space are very explicit. Regarding synthesis of newly assembling cuticle prior to ecdysis we show that preecdysal cuticle in mancas has mostly homogenous procuticle, with no distinct chitin-protein arrangement, although in certain regions a helicoidal arrangement of chitin-protein fibers is evident. In adult *Porcellio scaber* it is reported that several exocuticular lamellae are deposited in premolt stage (Ziegler 1997). Advanced stages of new cuticle formation in marsupial mancas need to be investigated in further research. Molting in two phases is typical in adult terrestrial isopods, while this is still not confirmed for developing marsupial mancas. In adult isopods some other morphological changes accompany molting process, particularly concerning calcium dynamics. In adult premolt stage ecdysal space in-between the old and new cuticle contains calcium storage granules (Ziegler 1994, Štrus and Blejec 2001). In mancas no similar granules were observed in the ecdysal space. In adults appearance of calcium deposits on the first four anterior sternites is a clear indication of the premolt stage (Steel 1982, Zidar et al. 1998). In our study calcium deposits in molting marsupial stages were not observed, indicating differences in calcium dynamics compared to adults. There are no data on calcium dynamics during marsupial development in terrestrial isopods.

Ultrastructural research of isopod egg envelopes and cuticle structure during ontogenetic development in a fluid-filled marsupium is a valuable approach to get insight into their differentiation and function and contribute to comparative analysis of ontogenetic development in different arthropods. Evaluation of the data on the newly forming cuticle structure and composition obtained in sequential phases of the ontogenetic development is important to get insight into the mechanisms of mineralized biological matrix assembly.

## Conclusions

During marsupial development of *Porcellio scaber* embryos and marsupial mancas in different stages are coated by different protective envelopes (Fig. 7).

Egg envelopes of isopod crustacean *Porcellio scaber* embryos are thinner and structurally less complex in comparison to egg envelopes of insect *Drosophila melanogaster.* These are expected differences due to different embryonic developmental strategies of these arthropods. Similarities in egg envelopes of these two species appear particularly in their inner egg envelope, the vitelline membrane.

Exoskeletal cuticles of *Porcellio scaber* prehatching late-stage embryo and marsupial mancas have already some features of the adult crustacean cuticle, but are significantly thinner. Three principal layers are distinguished, the outermost epicuticle, the middle exocuticle and the innermost endocuticle. Characteristic chitin-protein patterns of adult cuticle, particularly regarding the exocuticle, are not very distinct in prehatching late-stage embryo and become progressively more explicit in marsupial mancas. Cuticular scales and sensilla are fully elaborated already in prehatching late-stage embryo. In the distal portion of the exocuticle a dense layer is observed in marsupial mancas, which could correspond to the dense distal exocuticular layer of adult cuticle. Marsupial manca cuticle is not strongly calcified.

Cuticle renewal takes place already in prehatching late-stage embryo, where detachment of cuticle from hypodermis and partial disintegration of proximal endocuticle occur. Old cuticle detachment and new cuticle assembly appear in marsupial mancas of several stages. Morphological changes, related to calcium storage during molt cycle in adult isopods, were not observed in premolt marsupial stages.
